# Supplementary material for: Intensive Care Risk Estimation in COVID-19 Pneumonia Based on Clinical and Imaging Parameters: Experiences from the Munich Cohort
Source: J Clin Med. 2020 May 18;9(5):1514. doi: 10.3390/jcm9051514 (PMC7291055; doi:10.3390/jcm9051514)
Supplement: Supplementary file 1 [file jcm-09-01514-s001.zip › jcm-794247-supplementary.docx]

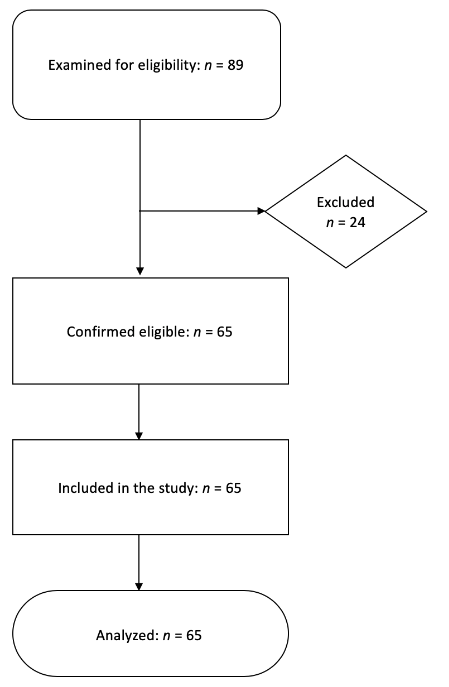


**Figure S1.** STROBE checklist and patient recruitment flow chart. Reasons for exclusion: (1) No CT chest available (*n* = 9); (2) No proof of SARS-CoV-2 in PCR (*n* = 15). CT, computed tomography; SARS-CoV-2, severe acute respiratory syndrome coronavirus 2; PCR, polymerase chain reaction.
